# The Role of Ankle Proprioception for Balance Control in relation to Sports Performance and Injury

**DOI:** 10.1155/2015/842804

**Published:** 2015-10-25

**Authors:** Jia Han, Judith Anson, Gordon Waddington, Roger Adams, Yu Liu

**Affiliations:** ^1^School of Kinesiology, Shanghai University of Sport, Shanghai 200438, China; ^2^Research Institute for Sport and Exercise, University of Canberra, Canberra, ACT 2600, Australia

## Abstract

Balance control improvement is one of the most important goals in sports and exercise. Better balance is strongly positively associated with enhanced athletic performance and negatively associated with lower limb sports injuries. Proprioception plays an essential role in balance control, and ankle proprioception is arguably the most important. This paper reviews ankle proprioception and explores synergies with balance control, specifically in a sporting context. Central processing of ankle proprioceptive information, along with other sensory information, enables integration for balance control. When assessing ankle proprioception, the most generalizable findings arise from methods that are ecologically valid, allow proprioceptive signals to be integrated with general vision in the central nervous system, and reflect the signal-in-noise nature of central processing. Ankle proprioceptive intervention concepts driven by such a central processing theory are further proposed and discussed for the improvement of balance control in sport.

## 1. Introduction

In many sports, superior balance ability is necessary to achieve the highest competitive level and avoid lower limb injuries [1–3]. To control balance, the central nervous system (CNS) integrates visual, vestibular, and proprioceptive information to produce motor commands that coordinate the activation patterns of muscles [4–6]. Proprioception has been defined as one's ability to integrate the sensory signals from various mechanoreceptors to thereby determine body position and movements in space [7, 8], and it plays a crucial role in balance control [5, 6, 9–11]. Theoretically, proprioceptive information from every part of the body contributes to balance control. This includes visual proprioception, as demonstrated by Lee and Aronson [12], although in sport the visual channel is often occupied with processing information about opponents or ball flight, so other proprioceptive sources are needed [13]. Sensory reweighting theory, which holds that the CNS can shift reliance to more reliable sources of information to optimize balance control [5, 6, 9] suggests that, for example, where vision is being used for tracking an activity in the external environment, the CNS may rely more on proprioceptive information from particular parts of the body for balance control. Ankle proprioception may be one of the more important components contributing to balance control in sport, because during most sports activities, the ankle-foot complex is the only part of the body contacting the ground. Ankle proprioception provides essential information to enable adjustment of ankle positions and movements of the upper body, in order to successfully perform the complex motor tasks required in elite sport [14, 15].

Ankle proprioception can be altered by general [16] and sport-specific training [17–19], sport-related injuries [20–25], and sport-induced fatigue [26, 27], all of which may subsequently lead to altered balance ability. The assessment of ankle proprioception in healthy individuals and subjects with musculoskeletal or neurological disorders has been addressed in three recent reviews [5, 7, 28]. The purpose of this review is to explore the association between ankle proprioception and balance control in a sporting context, and their roles in sport performance and sport injury. This provides an opportunity to determine the most appropriate methods for assessing ankle proprioception in athletes and potential central processing mechanisms underlying balance control. Understanding the mechanism underlying ankle proprioception and balance control may foreshadow optimal interventions to improve balance control in sport.

## 2. Balance Control and Ankle Proprioception in Sports Performance

Balance ability and ankle proprioception are both related to competition level in a range of sports. A systematic review on balance ability and athletic performance found that static balance ability of rifle shooters and archers was associated with their shooting accuracy, and dynamic balance ability of ice hockey players displayed a significant relationship with maximum skating speed [3]. In addition, a recent study investigating balance ability of a group of athletes from soccer, handball, basketball, and volleyball found that the balance ability of male athletes was significantly correlated with their agility performance [29]. This evidence suggests that balance control is fundamental to sports performance.

Similarly, ankle proprioception and sports performance are related. Han et al. [18] measured ankle proprioception of 100 elite athletes from 5 different sports—aerobic gymnastics, soccer, swimming, badminton, and sports dancing—and found that ankle proprioception scores were significantly predictive of sport performance level, extending up to Olympic level. In a subsequent study [30], the authors assessed proprioception at the knee, spine, shoulder, and hand in addition to the ankle and found proprioception at the shoulder and spine were also significantly associated with competitive level in these elite athletes. Of these three critical body sites—the ankle, shoulder, and spine—ankle proprioception was correlated most strongly with sport competition level and was the most significant predictor of sports performance [30]. These findings highlighted the importance of ankle proprioception in sporting success.

Thus, although visual [1] and vestibular [31] functions play important roles in balance control in sport, ankle proprioception, within the proprioceptive system, appears to be the most critical for balance control contributing to sport performance.

## 3. Balance Control and Ankle Proprioception in Sports Injury

Both balance control and ankle proprioception are negatively associated with ankle injuries [2, 35]. The relationship between poor balance control and heightened injury risk was identified 30 years ago, when in 1984 Tropp et al. [36] found that ankle injuries were almost 4 times more prevalent in soccer players with poor balance in comparison to those with normal balance ability. Similarly, Watson [37] found hurdling athletes and Gaelic football players with poor balance had nearly twice as many ankle injuries relative to their counterparts with normal balance. In addition, balance ability was found to be significantly associated with ankle injury risk in both younger male and female basketball players [38]. A recent systematic review summarized the available evidence and suggested that poorer balance ability is an intrinsic factor associated with increased ankle injury risk [35].

Similar reports of the relationship between ankle proprioception and ankle injury risk are also noted in the literature. For example, a longitudinal study found ankle proprioception could predict ankle injuries in college basketball players [39]. In addition, basketball players with poorer ankle proprioception used an altered pattern of cocontraction of ankle plantarflexors and dorsiflexors, which in turn resulted in greater impact force at the moment of landing associated with higher risk of ankle injury [40]. Ankle proprioception is one of the intrinsic factors associated with ankle injury, as identified by Witchalls et al. in their systematic review [35].

Ankle injuries often lead to disruption of muscles and tendons with associated damage to inherent mechanoreceptors [5, 41], which detrimentally alter the quality of proprioceptive information required for balance control. Unrehabilitated, impaired ankle proprioception after ankle injury [20–25] can subsequently result in long-term deterioration of postural and balance control. Gymnasts, dancers, and military sportsmen with poorer ankle proprioception after injury demonstrate worse performance in both static and dynamic postural and balance control tasks [42–45]. In addition, the common motor program hypothesis [46] suggests that there will be bilateral impairments in ankle proprioception in both the injured and uninjured sides [22, 47]. This bilateral impairment is also evident in postural and balance performance relative to healthy controls [48].

These findings suggest that ankle proprioception is closely related to balance control in sport injuries, and balance ability may be significantly affected by impaired ankle proprioception after injuries.

## 4. Mechanisms Underlying Ankle Proprioception to Balance Control in Sport

Sensory noise and sensory information reweighting are two possible mechanisms for the optimal use of sensory information in balance control [5, 6, 9]. Both models highlight the role of central processing in balance control and may explain the importance of ankle proprioception for balance control in sport. For example, ankle proprioception is superior in gymnasts, sports dancers, badminton players, soccer players, and swimmers [18, 30, 49, 50], suggesting that through years of sport-specific practice, ankle proprioception may be processed more efficiently and reliably in the brain [7]. If the CNS uses a reweighting strategy relying on more reliable sources of information to optimize balance control [5, 6, 9], refined ankle proprioception, with its signal-to-noise ratio reduced through practice, could be one of these more reliable sources of proprioceptive information in particular sports.

In addition, the observation of bilateral deficits in both ankle proprioception and balance control after ankle injury [22, 47] favored a central motor program view of bilateral limb movement control [51]. The data indicate that a higher-order central mechanism may exist for proprioceptive information processing contributing to postural and balance control [7, 52]. Indeed, a recent brain imaging study suggested that beyond peripheral reflex mechanisms, central processing of proprioceptive signals from the foot-ankle complex is essential for postural and balance control [53].

If central processing of proprioceptive information links ankle proprioception and balance control, then this has implications for ankle proprioceptive assessment. It suggests that the most appropriate measurement technologies are those that are relevant to normal function and that encompass ecologically valid components related to balance function [7]. This issue is also important for determination of the optimum ankle proprioceptive intervention to improve balance control in sport.

## 5. Selection of Ankle Proprioceptive Assessment Method Relevant to Balance in Sport

Proprioception can be assessed using different technologies/methodologies [5, 7, 28, 54]. There are three main technologies/methodologies used for ankle proprioceptive assessment. These are thresholds to detection of passive motion (TTDPM) [55–57], joint position reproduction (JPR) [26, 58, 59], and active movement extent discrimination assessment (AMEDA) [17, 60, 61]. The advantages and disadvantages of these testing protocols have been discussed in a recent review paper [7]. The current review focuses on the selection of appropriate measurement techniques relevant to balance function.

**Figure 1:**
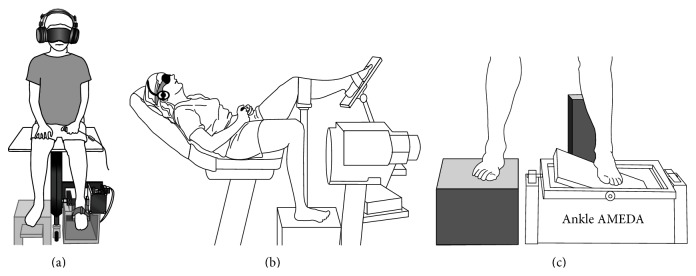
Examples of ankle proprioceptive assessment methods. (a) depicts the threshold to detection of passive motion (TTDPM) method, adapted from Yasuda et al. [32]; (b) depicts the joint position reproduction (JPR) method, adapted from Willems et al. [33]; and (c) depicts the active movement extent discrimination assessment (AMEDA) method, adapted from Symes et al. [34].

The three different technologies used for testing ankle proprioception are presented in Figure 1. It is clear from Figure 1 that some of the technologies adopt a nonweight bearing, either lying or sitting, position during testing and block both visual and audio information in order to assess “pure” ankle proprioception (Figures 1(a) and 1(b)). The ecological validity of these tests has been questioned however [62, 63] because the assessment conditions are markedly dissimilar to normal function and can therefore contribute little to understanding the role that ankle proprioception plays in balance control during sports and daily activities [7]. If central processing of ankle proprioception is crucial for balance control, ankle proprioceptive assessment should be conducted to reflect normal function that encompasses all proprioceptive signals arising from muscles, joints, and skin normally projected for integration in the CNS.

In a method designed to increase ecological validity, an upright, weight-bearing stance TTDPM option has been used, along with the AMEDA apparatus (Figure 1(c)), for ankle proprioception assessment [34, 56, 57, 64]. These techniques ensure activation of muscles, joint capsule compression, and skin stretch. More recently, Witchalls et al. [43, 65] have developed a “walk-across” AMEDA in order to assess dynamic ankle proprioception during normal gait, involving dynamic balance control.

In addition to ecological validity, assessment of ankle proprioception should acknowledge and incorporate the signal-noise nature of central processing [66–68]. It has been argued that when processing proprioceptive information, the brain has to deal with noise in the CNS arising from background or spontaneous neural activity [66, 69], which contributes to uncertainty in making decisions about positions and movements of a joint in space [7, 69]. Similarly, in balance control, the CNS has to process multiple sensory signals occurring against a noise background in order to determine body sway in space [6].

Waddington and Adams [70] applied signal detection theory (SDT) [71–73] to deal with noise-associated uncertainty in making judgments about ankle movements and positions. To do this, participants are required on any one trial to make an absolute judgment regarding one of five possible ankle movements. Nonparametric SDT Receiver Operating Characteristic (ROC) analysis [73] is then used to compare responses to pairs of ankle movements. The area under the ROC curve (AUC) [73] is used as the measurement of ankle proprioceptive sensitivity, representing a participant's ability to discriminate between the five ankle movements [7]. SDT gives a means to take an individual's uncertainty into account and produce an unbiased estimate of an individual's ankle proprioceptive performance [7].

In this way, the AMEDA technique both fulfills ecological validity and captures data in such a way as to address the signal-noise nature of central processing of proprioceptive information relevant to balance function. Although this method has not yet been used to determine the precise association between ankle proprioception measured with an AMEDA and balance ability in athletes, Guo et al. [56] has assessed ankle proprioception in an upright, weight-bearing stance using the TTDPM method and found that ankle proprioception can explain 53% and 44% of variance in anterior-posterior and medial-lateral posture sway directions, respectively.

## 6. Ankle Proprioceptive Intervention for Improvement of Balance Control in Sport

Both ankle proprioception and balance control are essential in sports [2, 3, 18, 40], and* passive* or* active* interventions to improve ankle proprioception and balance control, particularly after ankle injury, have been extensively reported in the literature [5, 11]. Regardless of the type of intervention—passive or active—central processing of ankle proprioception is likely to be critical for balance control. If this is the case, ankle proprioceptive intervention should focus on central processing mechanisms to improve balance control in order to enhance sports performance and minimize injuries.

### 6.1. Passive Intervention

A number of studies have explored effects of passive interventions, such as taping, bracing, compressing, or sport shoe insoles, on ankle proprioception [70, 74–78], with most finding passive techniques being not effective in improving ankle proprioception. Ankle taping and bracing, for example, are commonly used by athletes following a sprained ankle. Two systematic reviews consistently found ankle proprioception was not enhanced with the use of ankle tape or braces in athletes suffering repeated ankle sprains and with functional ankle instability [79, 80]. These findings suggest that ankle proprioception does not benefit from the restriction of ankle movement and/or from elastic resistance [81] imposed at the ankle joint.

In contrast, there is some evidence that the use of insoles, another passive intervention, has a positive effect on ankle proprioception in soccer players [70, 76]. It has been proposed that the use of textured insoles induces “essential noise” in the CNS, which in turn resulted in increased perception of information to support motor performance [82]. Consistent with this point of view, a systematic review found insoles with vibrating elements improved balance in older people [83]. However, not all types of insoles were found to be beneficial [83], presumably because only some proprioceptive signals combine with certain types of noise to enhance perception of proprioceptive information [82]. While ankle proprioception and balance control may be improved through a signal-noise resonance mechanism in the CNS [82], selection of appropriate techniques such as the height, texture, and vibration of particular insoles to optimize the signal-noise ratio currently needs further exploration if such design modifications are to be introduced into sports footwear.

### 6.2. Active Intervention

Various active exercise interventions, delivered in a task-specific paradigm, have been found to be effective for the improvement of ankle proprioception. It has been proposed that this occurs through neural mechanisms such as neural learning and neural plasticity [5]. Neural learning effects associated with ankle proprioception may be rapid. For example, Witchalls and colleagues [43] found athletes with chronic ankle instability improved their ankle proprioception in one session through ankle AMEDA test-retest practice. This improvement was thought to be due to central processing modifications. The authors [43] argue that mechanoreceptors at the ankle joint do not change during the repeated proprioceptive testing (the time is too short), and proprioceptive information originating from peripheral structures at the ankle does not significantly change either. Therefore, faster neural learning processes are likely to play the key role in improving ankle proprioception. Further research is needed to explore to what extent the improvement in ankle proprioception through neural learning translates to better balance control in the sporting context and whether such learning should be conducted as explicit or implicit learning [84].

In contrast, some neural changes may require weeks, months, or even years of practice. Several weeks of wobble-board training [17, 85, 86], Tai Chi exercise [56, 87, 88], and other specifically designed exercise programs [89–91] have been shown to improve ankle proprioception and balance control in athletes, university students, and older people, with or without ankle instability. Kiers et al. [92] argue that exercise on an unstable surface might not target ankle proprioception* per se* but rather trains the CNS to shift the weighting of sources of proprioceptive signals to improve balance. If this is the case, yachting and figure-skating athletes whose daily activities involve performing motor tasks on an unstable surface would benefit from exercise on a similar surface [93]. What is not known is whether combined exercise on both unstable and stable surfaces provides even greater benefits for ankle proprioception and balance control than just active training on unstable surfaces.

Apart from training surfaces, another issue associated with active ankle proprioceptive training is whether the training should focus on the injured side or should involve both sides after sports injury. Given a significant and positive correlation found between performance of both ankles in healthy and injured participants [22, 46, 61], ankle proprioceptive training should also involve the intact side [46, 61]. Some evidence suggests that motor skills are able to be transferred between hemispheres [94], indicating that training on the uninjured side could benefit the affected side. However, interhemispheric motor skill transfer may be affected by ageing [95] and limb dominance [96]. In addition, a recent study investigating sensory reweighting of proprioceptive information from each leg during balance control found that proprioceptive signals from each leg were weighted independently, and weighting of proprioceptive signals of one leg had no effect on the weight of the proprioceptive information of the other leg [9]. Taken together, proprioceptive training is likely to be most beneficial for improving ankle proprioception* per se* when conducted on each leg and by optimizing ankle proprioceptive information reweighting for balance control in sport. Future research is needed to elucidate the CNS process associated with active interventions.

### 6.3. Other Considerations

Although developing better proprioception and balance control through training is a common goal for athletes and there is mounting evidence suggesting that active interventions such as wobble board training aid in doing this, there may also be a significant genetic component to proprioceptive ability and balance control. This is likely to be more evident in elite athletes, who are striving to be the best of the best, where training levels are already extensive. In studies by Han et al. [18, 30], it was reported that ankle proprioception scores were not significantly correlated with years of training, suggesting that the amount of improvement in ankle proprioception associated with sports training may be constrained by biologically determined factors [30]. From studies of twins performing balance control tasks, it has been suggested that there may also be a genetic component contributing to balance control [97, 98]. If this is the case, in order to achieve the highest competitive level an athlete may also need to have genetic potential for better ankle proprioception and balance control. If correct, future sport talent identification may need to consider natural aptitude in both ankle proprioception and balance ability when selecting potential elite athletes.

## 7. Conclusion

Proprioception plays an essential role in balance control, and ankle proprioception is arguably the most important aspect of this. Central processing of ankle proprioceptive information, along with other sensory information, enables integration for postural and balance control. When assessing ankle proprioception for generalization to applied situations, the method used should have ecological validity and allow proprioceptive signals to be integrated in the central nervous system, in order to reflect the signal-noise nature of central processing in sports activities. In addition, ankle proprioceptive interventions, passive or active, should therefore be predicated on discriminating signal from noise in central processing, to attain optimal outcomes.
